# Mechanistic target of rapamycin inhibitors: successes and challenges as cancer therapeutics

**DOI:** 10.20517/cdr.2019.87

**Published:** 2019-12-19

**Authors:** Muireann Ní Bhaoighill, Elaine A. Dunlop

**Affiliations:** Division of Cancer and Genetics, Cardiff University, Cardiff, CF14 4XN, UK.

**Keywords:** Mechanistic target of rapamycin, cancer, rapamycin, rapalog, clinical trial, resistance

## Abstract

Delineating the contributions of specific cell signalling cascades to the development and maintenance of tumours has greatly informed our understanding of tumorigenesis and has advanced the modern era of targeted cancer therapy. It has been revealed that one of the key pathways regulating cell growth, the phosphatidylinositol 3-kinase/mechanistic target of rapamycin (PI3K/mTOR) signalling axis, is commonly dysregulated in cancer. With a specific, well-tolerated inhibitor of mTOR available, the impact of inhibiting this pathway at the level of mTOR has been tested clinically. This review highlights some of the promising results seen with mTOR inhibitors in the clinic and assesses some of the challenges that remain in predicting patient outcome following mTOR-targeted therapy.

## Introduction

The mechanistic target of rapamycin (mTOR) is a 289-kDa serine/threonine protein kinase that belongs to the class IV phosphoinositide 3-kinase (PI3K)-related kinase (PIKK) family. Considered a principal regulator of cell growth and metabolism, this multifunctional kinase coordinates upstream signals from growth factors, and nutrient and oxygen resources to control cell growth in both physiological and pathological settings. mTOR does this through regulating fundamental biological activities including cell proliferation and survival, cell cycle, protein synthesis, and glucose metabolism^[1]^.

mTOR functions as a central catalytic subunit to two complexes, mTOR complex 1 (mTORC1) and 2 (mTORC2). mTORC1 and mTORC2 are structurally and functionally different, are stimulated by different mechanisms and play different roles in controlling cell bioactivity^[2]^. They also express individual substrate specificity.

mTORC1 signalling is responsive to extracellular stimulation via growth factor, amino acid and energy resources in order to stimulate protein biosynthesis, cell proliferation and angiogenesis. At its core, mTORC1 is comprised of mTOR, regulatory-associated protein of mTOR (RAPTOR) and mammalian lethal with Sec13 protein 8 (mLST8)^[1]^. Other proteins also associate with mTORC1, depending on species and specific conditions, including proline-rich AKT Substrate 40 (PRAS40)^[3]^, DEP domain-containing mTOR interacting protein (DEPTOR)^[4]^, GRP58^[5]^, telomere maintenance 2 (TEL2)-interacting protein 1 (TTI1), TEL2^[6]^ and Ras family small GTPase 1 (Rac1)^[7]^.

mTORC2 is largely stimulated by upstream growth factor binding. This complex is composed of mTOR, rapamycin-insensitive companion of mTOR (RICTOR), mLST8, proline-rich protein 5 (PRR5, also known as Protor-1), heat shock protein 70, DEPTOR, GRP58, TTI1-TEL2, Rac1, mammalian stress-activated protein kinase interacting protein 1 and protein observed with RICTOR (PROTOR). mTORC2 plays key roles in cytoskeletal organisation and facilitating cell survival by its activation of multiple related due to sequence similarity (AGC) family kinases, including serum and glucocorticoid kinase, protein kinase B (AKT), and protein kinase C^[8]^.

## Cellular processes controlled by mTORC1

**Figure 1:**
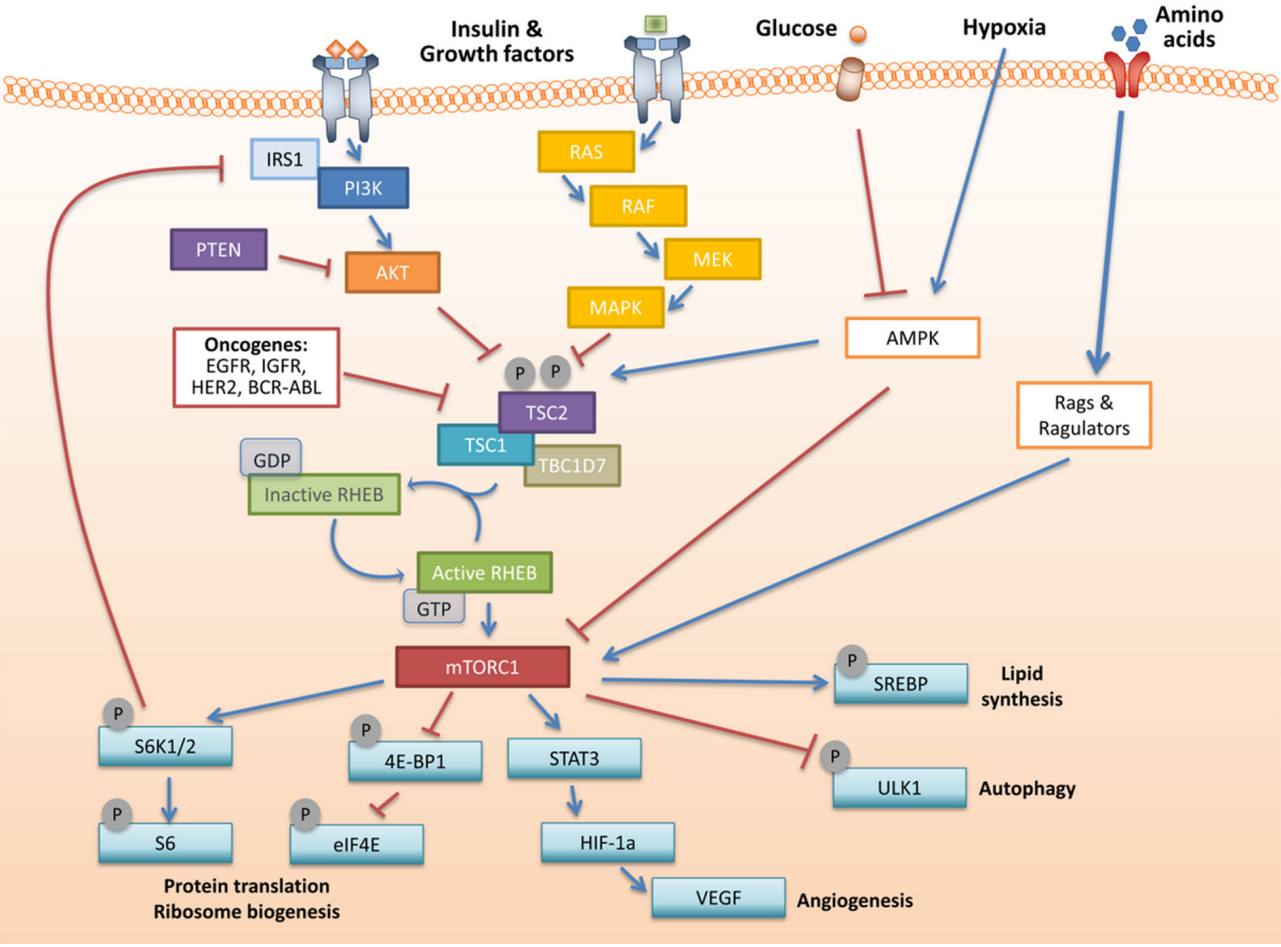
An overview of the mTORC1 signalling pathway. IRS1: insulin receptor substrate 1; PI3K: phosphoinositide 3-kinase; PTEN: phosphatase and TENsin homolog; AKT: AKT8 virus oncogene cellular homolog; AMPK: AMP-activated protein kinase; TSC1: tuberous sclerosis complex 1; TSC2: tuberous sclerosis complex 2; RHEB: ras homolog enriched in brain; GDP: guanosine diphosphate; GTP: guanosine triphophate; mTORC1: mammalian target of rapamycin complex 1; S6K1/2: ribosomal protein S6 kinase 1/2; 4E-BP1: eIF4E binding protein 1; eIF4E: eukaryotic initiation factor 4E; STAT3: signal transducer and activator of transcription 3; HIF-1α: hypoxia inducible factor 1 alpha; VEGF: vascular endothelial growth factor; SREBP: sterol regulatory element-binding protein; ULK1: Unc-51-like autophagy activating kinase 1

Both complexes incorporating mTOR play distinct physiological roles, but we only provide a brief overview of mTORC1 signalling in this review. mTORC1 signalling is promoted by growth factors and amino acid sufficiency. Growth factor or insulin binding to receptors at the cell surface triggers PI3K/AKT signalling pathway activation^[9,10]^. Once activated, AKT phosphorylates tuberous sclerosis complex 2 (TSC2) protein, inducing its dissociation from the tumour suppressor complex it forms with TSC1^[11]^. Dissociation of this heterodimer TSC1/2 complex causes loss of GTPase activity of the complex. Thus, its inhibitory effect over Ras homolog enriched in brain (RHEB) ceases, allowing the activation of mTORC1 signalling on lysosomes^[12]^ [Figure 1]. Additionally, insulin-activated AKT phosphorylates mTORC1-associated PRAS40, causing its dissociation from RAPTOR, which serves as a means to activate mTORC1 signalling independently of TSC1/2^[3]^. These upstream signalling mechanisms are reviewed in more detail in ref.^[13]^. Amino acid sufficiency is sensed by the Rag GTPases and the Ragulator complex and is required for full activity of mTORC1. Amino acid sensing by mTORC1 has recently been comprehensively reviewed in ref.^[14]^.

Once activated, mTORC1 signalling drives increases in cell size and proliferation. To achieve this, mTORC1 signals to important downstream effectors, p70S6K1 and 4E-BP1. Both of these proteins express a common mTORC1 signalling motif, permitting their respective recognition by RAPTOR and their subsequent phosphorylation by activated mTORC1^[15]^. Their phosphorylation enhances protein synthesis through two means. Hypophosphorylated 4E binding proteins bind tightly to eukaryotic translation initiation factor (eIF4E) at the 5’ cap of mRNA, preventing interaction with eIF4G and thus preventing translation. Once phosphorylated at multiple sites by mTORC1, 4E-BP1 releases from eIF4E, allowing eIF4G and eIF4A to bind free eIF4E (collectively forming the eIF4F complex) at the 5’ cap of mRNA, thus increasing mRNA translation, including proteins necessary for G1-to-S phase transition^[16]^. Enhanced protein translation is also facilitated when p70S6K1 becomes phosphorylated at its Thr389 site by activated mTORC1. Through a variety of pathways, this controls effective ribosomal biogenesis, thereby potentiating translation^[13]^.

In addition to protein synthesis, mTORC1 activation controls several other growth promoting processes. When active, mTORC1 induces the upregulation of hypoxia-inducible factor-1 alpha (HIF-1α) and vascular endothelial growth factor (VEGF) to promote both angiogenic potential^[17]^ and an increase in glycolysis^[18]^. mTORC1 also positively regulates glutamine metabolism by inhibiting sirtuin 4 (SIRT4)^[19]^, as well as facilitating lipid synthesis and influencing the pentose phosphate pathway through its action on sterol regulatory element binding protein 1 (SREBP-1)^[18,20,21]^. mTORC1 also cross-talks with autophagy through ULK1 to maintain a homeostatic balance between anabolic and catabolic processes^[22]^.

## mTOR inhibitors

### The discovery of rapamycin and its early use

Rapamycin, known clinically as sirolimus, is a natural macrolide isolated originally from soil samples of Rapa Nui (Easter Island) by Sehgal and colleagues^[23]^. Produced by *Streptomyces hygroscopicus*, it was discovered serendipitously in a screen for antimicrobial activity against *Candida albicans*. It was initially recognised for its potent anti-fungal activity, but was later shown to be a potent immunosuppressant and anti-proliferation agent. Today, rapamycin is used clinically to prevent kidney and cardiac graft rejection in post-transplantation patients^[24,25]^. However, it is its role as an anti-proliferative agent that is the focus of this review.

Functional studies on the mechanism of action of rapamycin identified the cellular target of rapamycin, named TOR. Rapamycin binds to immunophilin 12-kDa protein FKBP12, and this drug-protein complex causes contraction of the mTORC1 dimer active site cleft from 20 Å to 10 Å^[26,27]^, thus inhibiting mTORC1 activity selectively and allosterically.

### The development of rapalogs

**Figure 2:**
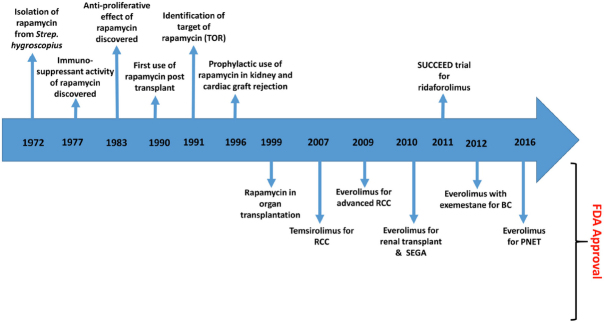
Timeline of rapamycin and rapalogs from discovery to clinic. Rapalogs include temsirolimus, everolimus and ridaforolimus. RCC: renal cell carcinoma; SEGA: subependymal giant cell astrocytoma; BC: breast cancer; PNET: pancreatic neuroendocrine tumours; FDA: Food and Drug Administration (United States of America)

The development of rapamycin analogues, termed rapalogs, began in an effort to improve the solubility, stability and poor pharmacokinetic characteristics of rapamycin [Figure 2]. First generation rapalogs included temsirolimus (CCI-779), everolimus (RAD001) and ridaforolimus (AP23573). Rapalogs elicit the same mechanism of action as rapamycin. They bind to FKBP12 and complex with the FKBP12-rapamycin binding (FRB) domain on mTOR, constricting its site of action and thus inhibiting substrate docking and phosphorylation^[28]^. As with rapamycin, their downstream effects include inactivation of p70S6K1 and 4E-BP1, preventing ribosomal biogenesis and mRNA translation^[29]^. The rapalogs inhibit cell cycle progression by arresting cells at the G1-S interface, thereby achieving anti-proliferative effects.

Despite the efforts to improve pharmacokinetics using rapalogs, bioavailability is still considered a limitation of mTOR inhibitors. For example, everolimus has a bioavailability of 16% *vs.* that of 10% for sirolimus, as determined in an *in vivo* model^[30]^. Furthermore, rapamycin and rapalogs have therapeutic limitations given their inability to inhibit mTORC1 signalling completely, due to activation of compensatory pathways (discussed in more detail in “Pitfalls of mTOR inhibitor use in the clinic” section) and their differential inhibitory effects on 4E-BP1 *vs.* S6Ks^[31]^. To overcome this, ATP-competitive inhibitors of mTOR were developed, which inhibit both mTORC1 and mTORC2. These showed great potential in pre-clinical studies, demonstrating high potency and selectivity for mTOR inhibition and suppressing cell growth and survival^[32-34]^. They are currently being evaluated in clinical trials^[35,36]^.

## mTOR and cancer

### mTOR signalling in cancer

With its key role in regulating of a number of anabolic cellular processes, it is unsurprising that cancer cells hijack the mTORC1 pathway as part of tumour development and growth. Aberrant activation of mTORC1 signalling permits the cancer cells to sustain their proliferative drive even when nutrients and growth factor stimulation are lacking.

mTORC1 pathway activation has been reported in a wide variety of solid tumours and commonly occurs due to aberrant expression of upstream regulators of the pathway. For example, the tumour suppressor, phosphate and tensin homolog deleted from chromosome ten (PTEN), regulates the PI3K/AKT pathway which converges on mTOR [Figure 1]. PTEN is lost or mutated in at least seven cancer types, including breast and prostate cancers^[37]^. The mitogen-activated protein kinase (MAPK) pathway can also cross-talk with mTORC1 signalling [Figure 1], through phosphorylation of TSC2^[38]^ and RAPTOR^[39]^. The RAS and RAF components of the pathway are frequently mutated in cancer, with gain-of-function missense mutations in RAS genes detected in around 25% of human cancers^[40]^. Activating BRAF mutations are found in around half of melanomas and 60% of thyroid cancers^[41]^. Upstream amplification of growth factor receptors and substrates, such as epidermal growth factor receptor (EGFR), insulin-like growth factor receptor and insulin receptor substrate (IRS), promotes the PI3K and MAPK pathways, which cross-talk with mTORC1, and thus enhance tumour-promoting processes.

Less commonly, mTORC1 activation can occur through mutations to the mTORC1 pathway components themselves, such as TSC1, TSC2 and mTOR. Loss of function mutations of the tumour suppressor TSC1 are seen in bladder cancer^[42]^, while 25 different TSC2 mutation sites, mainly in or near the RAP-GAP domain, have been reported in pancreatic neuroendocrine tumours (PNET)^[43]^. TSC2 mutations are also found in hepatocellular carcinoma^[44]^. A recent study analysing The Cancer Genome Atlas (TCGA) pan-cancer cohort looked in detail at molecular alterations involving the PI3K/AKT/mTOR pathway^[45]^. In their pan-cancer analyses, the authors reported 2% mutation rate in TSC1 and 4% mutation rate in mTOR. Additionally, some cases showed TSC1 or TSC2 rearrangements^[45]^. Various mTOR mutations were found in another study which used partial genome sequencing data from the TCGA, CCLE, International Cancer Genome Consortium (ICGC), and Catalogue of Somatic Mutations in Cancer (COSMIC) databases. These authors reported that, when normalised for gene length, mTOR had the highest percentage of recurring mutations out of all the mTOR pathway genes, and they identified 33 novel mTOR pathway-activating mutations^[46]^.

### The promise of mTOR inhibitors in cancer

Due to the major involvement of mTORC1 signalling in cancer described above, drugs which bind mTORC1 selectively and specifically were anticipated to hinder cancer cell metabolism and downstream protein and lipid synthesis, thereby eliciting anti-cancer effects. In the hamartoma syndrome, tuberous sclerosis complex (TSC), loss of function of TSC1 or TSC2 leads to mTORC1 hyperactivation and the formation of cysts and tumours in multiple organs. In this condition, where the defined genetic cause of mTORC1 activation is known, rapamycin and rapalogs have shown great promise. Kidney cysts, known as angiomyolipomas, regress while on therapy^[47-49]^, while there is also an improvement in neurological outcomes, with a marked reduction in the volume of subependymal giant-cell astrocytomas and seizure frequency^[50-52]^. Recently, benefits from mTOR inhibitors were also reported for cardiac rhabdomyomas, arrhythmias and neurological outcomes in very young TSC patients^[53]^.

With the frequency of dysregulation of mTORC1 signalling observed in cancer, it might be expected that reductions in tumour volume similar to that seen in TSC would be apparent in sporadic cancers following rapamycin treatment. In renal cell carcinoma (RCC), phosphorylated ribosomal protein S6, a marker of mTORC1 activation, was found to stain strongly in 85% of RCC tissues examined^[54]^. In accordance with this finding, the RECORD-1 randomised, Phase III study of the rapalog, everolimus, in metastatic RCC found a reduction in the risk of disease progression or death relative to placebo. This positive result prompted an early termination of this study, with patients permitted to cross from the placebo to the everolimus study arms^[55,56]^. While progression-free survival was improved with everolimus, the tumour volume response was low and follow-up analysis indicated that this may be partly due to resistant lesions that continue to grow despite everolimus treatment^[57]^. The large prospective Phase II RAPTOR study in papillary metastatic RCC also demonstrated some clinical benefit of everolimus treatment for this patient population^[58]^.

The rare PNET have also been found to have mTOR hyperactivity, with one study demonstrating that 85% of primary tumours had altered protein levels of the tumour suppressors TSC2 and PTEN^[59]^. The authors found that mTORC1 activity in PNET cell lines could be inhibited by rapamycin^[59]^. Several studies have now evaluated the clinical impact of mTOR inhibitors in PNET. In RADIANT-1, which assessed everolimus in advanced PNET, everolimus, with or without concomitant octreotide long-acting release, demonstrated antitumor activity, with 59.3% of patients on everolimus monotherapy showing tumour shrinkage. Overall survival also compared favourably with large institutional series^[60]^. A further Phase III study (RADIANT-3) showed that everolimus, as compared with placebo, significantly prolonged progression-free survival among patients with progressive advanced PNET^[61]^. As with the RCC trials, tumour shrinkage was only seen in a small proportion of patients, so the benefit from everolimus treatment was seen primarily in the stabilisation of disease or minor tumour shrinkage and in the lower incidence of progressive disease^[61]^. These positive findings about prolonged progression-free survival in PNET patients on everolimus treatment were further supported by the RADIANT-4 study^[62]^ and everolimus has now been approved worldwide for the treatment of patients with advanced, progressive, well-differentiated, non-functional PNET^[63]^.

mTOR inhibitors as monotherapy have also shown some clinical activity in biliary tract cancer (BTC). In an exploratory Phase II trial, favourable anti-tumour activity was observed in a subset of patients with advanced BTC, although the study narrowly failed to meet its primary endpoint of disease control rate (RADiChol Study, NCT00973713)^[64]^. Additionally, the use of rapalogs as a maintenance therapy is being explored. For example, ridaforolimus (AP23573) is under investigation as a maintenance therapy for metastatic soft tissue or bone sarcomas in patients with stable disease or improved disease following four cycles of chemotherapy. In the SUCCEED trial (NCT00538239), this rapalog induced a 28% reduction in the risk of disease progression in this patient cohort^[65]^, showing sustained anti-proliferative effects of ridaforolimus. It is thought that, while statistically significant, the absolute magnitude of clinical disease control may have been small due to the innate aggressiveness of this disease.

From this evidence, it appears that rapalogs, used as single agents, have the potential to reduce tumour volume and improve patient care in some conditions. However, despite the common activation of mTORC1 signalling in cancer, trials with rapamycin and its analogues as monotherapies in many cancer types have not been as effective as had been hoped [Table 1].

**Table 1:** A summary of the clinical successes and failures of rapalog treatment

	Cancer/Disease type	Study and reference
Food and Drug Administration approved	Renal cell carcinoma	RECORD-1 trial^[55]^; [56,113]
	HR+, HER2- breast cancer	[118]; TAMRAD^[119]^; BOLERO-2^[120]^; PrE0102^[121]^
	Pancreatic neuroendocrine tumours	RADIANT-1 (NCT00363051)^[60]^; RADIANT-3 (NCT00510068)^[61]^; RADIANT-4 (NCT01524783)^[62,63]^
	Tuberous sclerosis complex	NCT00457808^[47]^; NCT00490789^[48,49]^; NCT00411619^[50]^; NCT00126672^[51]^; EXIST-1 (NCT00789828)^[52]^; [53]
Potential clinical benefit	Biliary tract cancer	RADiChol study (NCT00973713)^[64]^; EUDRACT 2008-007152-94^[125]^
	Thyroid cancer (in patients refractory to other agents)	NCT01263951^[126]^; NCT00936858^[127]^
No clinical benefit seen	Gastric cancer	GRANITE-1, NCT00879333^[67]^
	Non-small-cell lung carcinoma	[68-70]
	Metastatic colorectal cancer	[71,73]
	Prostate cancer	[74,75]

### Pitfalls of mTOR inhibitor use in the clinic

Many studies demonstrate that the initial promise of rapalog therapy has not been realised. A common pattern seen in trial data is of a modest response to rapalog monotherapy which does not lead to a significant improvement in patient outcomes. For example, while everolimus monotherapy initially looked promising in a Phase II gastric cancer study, with a decrease in tumour size from baseline observed in 45% of patients^[66]^, a follow up Phase III study (GRANITE-1) showed everolimus did not significantly improve overall survival compared with best supportive care^[67]^. A Phase I trial of everolimus in solid tumours indicated that some non-small-cell lung cancer (NSCLC) patients could achieve a partial response to therapy^[68]^. However, another small study using temsirolimus as a single agent in NSCLC demonstrated clinical benefit but did not meet the primary objective of confirmed response rate^[69]^. A Phase II trial which looked for a benefit of adding everolimus to erlotinib treatment did not lead to any substantial improvement in NSCLC disease stabilisation^[70]^.

In colorectal cancer, 40%-60% of tumours show mTOR activation^[71]^ and everolimus showed some efficacy in patients with metastatic colorectal cancer in Phase I studies^[68,72]^. However, in a Phase II study which enrolled 199 heavily pre-treated metastatic colorectal cancer patients who had progressive disease, single-agent everolimus showed minimal activity, with disease stabilisation the best response seen^[71]^. Supporting this, a second Phase II study in metastatic colorectal cancer was unable to demonstrate a clinically meaningful anti-tumour activity of temsirolimus^[73]^. Although stabilisation of disease was seen in a proportion of patients, no objective responses according to RECIST criteria were observed^[73]^.

Rapalogs have also been trialled in prostate cancer, where it was hoped that the high level of PI3K-mTORC1 signalling, often due to PTEN loss, would make these tumours susceptible to mTORC1 inhibition. However, the studies to date have not shown sufficient clinical activity of rapalogs as monotherapy. One Phase II trial of temsirolimus in men with castration-resistant metastatic prostate cancer was stopped prematurely because of a lack of efficacy^[74]^. Another Phase II trial of everolimus in a chemotherapy-naïve metastatic castration-resistant prostate cancer population revealed that mTORC1 inhibition in unselected patients only had a moderate effect^[75]^.

### Reasons for rapalog resistance

It is not immediately clear why rapalogs have not lived up to their expected promise in many solid cancers, although several suggestions have been put forward. One of the likely reasons is that it is caused by reactivation of mTORC1-regulated negative feedback loops. For example, active mTORC1 phosphorylates S6K1, which in turn promotes degradation of IRS-1 leading to downregulation of PI3K/AKT signalling. Following mTORC1 inhibition by rapamycin, IRS-1 expression is induced and the repression is relieved, thus reactivating PI3K signalling and promoting cell growth and survival^[76]^. A mTORC1-MAPK feedback loop has also been identified, with mTORC1 inhibition inducing MAPK cascade activation in tumour samples taken from patients treated with everolimus. Interestingly, mTORC1 inhibition seemed to elicit a differential MAPK activation depending on the specific dose and administration schedule of everolimus^[77]^. Both PI3K/AKT and MAPK signalling are also regulated by the mTORC1 substrate, growth factor receptor-bound protein 10 (Grb10). mTORC1-mediated phosphorylation stabilises Grb10, leading to negative regulation of PI3K/AKT and MAPK signalling. Rapamycin treatment relieves this feedback inhibition as Grb10 becomes destabilised^[78,79]^.

mTORC1 activity also cross-talks with, as well as negatively regulates, the catabolic process of autophagy. While rapamycin treatment is often less effective at re-activating autophagy in mammalian cells than in yeast^[80]^, mTORC1 inhibition could permit some reactivation of autophagy in cancer cells, thus allowing them to recycle macromolecules more effectively, maintain homeostasis and survive. In addition to the incomplete re-activation of autophagy, rapamycin treatment also incompletely inhibits protein synthesis, due to differential regulation of S6K1 and 4E-BP1 phosphorylation^[31]^. Furthermore, due to similar architecture, other AGC kinase family members such as p90 ribosomal S6 kinase (RSK), can compensate for S6K1 inhibition^[81]^. As RSK family members are activated via classical MAPK signalling, they are largely unaffected by rapamycin and so can permit rpS6 phosphorylation and translation initiation in an mTORC1-independent manner^[81]^.

A further pitfall of rapalogs is their cytostatic mode of action. This has been clearly shown during trials in TSC, where cessation of everolimus treatment permitted rapid regrowth of angiomyolipomas^[47]^. This weakness of rapalogs has similarly been seen in sporadic cancer, where stabilisation of disease, rather than partial or complete remission, is often the best response for many patients^[61,71]^.

Additionally, it is common for treatment-resistant cancer cells to have acquired secondary mutations in the kinase being targeted. Some mTOR inhibitor-naïve tumours have been reported to contain mTOR mutations^[82,83]^, but mTOR has also been reported to become mutated in cancers treated with mTOR inhibitors. An anaplastic thyroid cancer patient demonstrated a sustained 18-month response to everolimus, with a substantial decrease in tumour size. The original tumour cells were found to contain an inactivating mutation in TSC2, explaining the acute sensitivity of the tumour to mTORC1 inhibition. However, resistance to treatment developed. Although the TSC2 mutation was still present in the resistant cells, a new mTOR mutation was also detected in these cells. This mutation was within the FRB domain and was shown to confer rapamycin resistance in cell culture experiments^[84]^. Mutations within the wider mTORC1 signalling network have also been proposed to contribute to rapamycin resistance. Epigenetic silencing of the PP2A regulatory B55b subunit (PPP2R2B) has been reported in over 90% of colorectal cancers^[85]^. Through mechanistic analysis, the authors proposed that loss of PPP2R2B resulted in PDK1-dependent myc phosphorylation in response to rapamycin, resulting in rapamycin resistance^[85]^.

Tumour heterogeneity may be another contributing factor to rapalog resistance. Bulk tumours may comprise of groups of cells with differing sensitivities to treatment due to the presence of distinct molecular signatures. With the development of powerful genomic profiling techniques, researchers have been able to probe the genetic heterogeneity of tumours. An interesting study used exon-capture multiregion sequencing to profile spatially separated samples from a renal cell carcinoma^[86]^. Amongst the 101 non-synonymous point mutations identified across the different tumour regions, they reported a heterogeneous mTOR mutation which rendered the kinase constitutively active. The regions containing this mutation corresponded to the areas with increased S6 phosphorylation^[86]^. This identification of spatially-separated somatic mutations, which can alter signalling activity, suggests that single tumour-biopsy specimens will only reveal a minority of the genetic variation present in a tumour and so could be poor predictors of treatment response.

## Overcoming resistance

### Strategies to improve rapalog efficacy

What can be done to overcome these issues? There are two main approaches that can be taken to enhance the efficacy of rapalogs in the clinic. The first is to use them in combined therapies. For example, in an effort to overcome the feedback loop to autophagy, mTOR inhibitors have been combined with the autophagy inhibitor, hydroxychloroquine. This has been shown to be safely tolerated in patients with lymphangioleiomyomatosis, with the higher dose level of hydroxychloroquine showing potential for a durable effect following treatment cessation^[87]^. The combination of everolimus and hydroxychloroquine is also promising in renal cell carcinoma, where a Phase I trial showed disease control in 67% of evaluable patients^[88]^, although a trial in soft tissue sarcoma was less promising^[89]^. Ongoing and completed trials are testing autophagy and mTORC1 inhibition in breast cancer (NCT03032406), advanced malignancies (NCT01266057 and NCT00909831) and myeloma (NCT01396200).

To counteract the re-activation of Akt and MAPK signalling following mTORC1 inhibition, dual PI3K/mTOR inhibitors and combination therapies of mTOR and MEK inhibitors have been trialled. As the hinge region of mTOR shares high sequence homology with that of PI3K^[90]^, compounds originally designed to target PI3K also show binding affinity for mTOR. These ATP-competitive inhibitors compete for the ATP binding pocket on both mTOR and PI3K kinases, and it was hoped that a broader anti-cancer effect could be achieved with this dual targeting. A number of dual PI3K/mTOR inhibitors looked very promising in the pre-clinical stage. However, results from clinical trials have been mixed. In PNET patients, the dual inhibitor BEZ235 was poorly tolerated. Although there was some evidence of disease stability, the study did not proceed to the second stage^[91]^. A similar outcome was seen in a multicentre Phase I/Ib trial in advanced solid tumours, including breast cancer, where PI3K/mTOR inhibition by BEZ235 was not sufficient to achieve an adequate antitumor effect with a favourable safety profile^[92]^. Limitations were also seen with another dual inhibitor, PF-04691502 when tested in a variety of solid cancers. Although there was evidence of PI3K down-regulation, no evaluable tumour responses were seen^[93]^. However, some more encouraging results of this approach have been observed. Using the dual inhibitor LY3023414, more than half of the advanced cancer patients treated at or above the maximum tolerated dose in a Phase I study demonstrated a decrease in the sum of target lesions. Interestingly, one endometrial cancer patient had a partial response that lasted for over 18 months. This patient was found to have a PIK3R1 mutation and a truncating PTEN mutation^[94]^.

A very similar story emerges with mTOR and MEK inhibition. Pre-clinical data are promising^[95]^, but clinical trials have shown the combination is not very well tolerated^[96,97]^. Although some patients have shown partial responses and stable disease, the overall clinical activity of the trametinib and everolimus combination in patients with advanced solid tumours is modest, with a dose providing acceptable tolerability and adequate drug exposure not achievable^[96]^.

Other trials have combined rapalogs with conventional chemotherapy drugs. As with the studies combining mTOR with PI3K or MEK inhibitors, these have often proved disappointing. In high-risk acute myeloid leukaemia patients, everolimus combined with mitoxantrone, etoposide and cytarabine showed substantial clinical activity and was reported as an effective, well-tolerated regimen for salvage or initial therapy of high-risk acute myeloid leukaemia^[98]^. However, the UK NCRI AML17 trial, where patients received everolimus sequentially with chemotherapy, reported no clinical benefit^[99]^. A Phase II trial of sirolimus with cyclophosphamide in sarcoma found that progression-free survival was similar to other active treatment regimens^[100]^. In clear cell RCC, pre-clinical trials suggested synergy between histone deacetylase inhibitors and mTOR inhibitors. Phase I data show this combination was tolerated, although no apparent clinical benefit was seen in the advanced clear cell RCC cohort under study^[101]^. Anti-VEGF therapy in combination with everolimus was tested in a Phase II trial of recurrent ovarian, fallopian tube or peritoneal carcinoma but the combination did not significantly prolong progression-free survival compared to bevacizumab alone^[102]^. More promising were two case reports in the pancreatic cancer setting which indicated that the addition of sirolimus to a gemcitabine, capecitabine and docetaxel regimen resulted in tumour regression^[103]^. Modest efficacy was also reported in a recent Phase I trial in triple negative breast cancer, which combined everolimus with the cell cycle inhibitor, eribulin. Triple negative breast cancer has poor clinical outcomes and more precise and personalised treatments are needed. This combination was shown to be safe, with a disease response rate of 36%^[104]^.

The second option is to molecularly stratify patients to only treat those likely to benefit from rapalogs based on the genetics of their cancer. This strategy is supported by case reports of patients who have demonstrated marked and durable responses to rapalog monotherapy. For example, next generation sequencing analysis of a liquid biopsy from a patient with advanced breast cancer, who failed to respond to conventional chemotherapy, showed mutations in PIK3CA, PTEN and mTOR. Everolimus monotherapy resulted in partial remission after two months and sustained stable disease after 18 months^[105]^. Similarly, a near-complete and sustained response to everolimus was also reported in a metastatic breast cancer patient with a STK11 point mutation^[106]^. A durable and ongoing complete response to everolimus was also reported in a patient with metastatic bladder cancer, even though the trial that they were enrolled in (NCT00805129) failed to achieve its progression-free survival end point. This super-responder was found to have a TSC1 mutation^[107]^.

There are also indications from larger trials that selecting only patients with mTORC1 signalling dysregulation for rapalog therapy could be an effective strategy. A small Phase II trial of everolimus as a single agent in castration-resistant prostate cancer (mCRPC) showed that, although everolimus activity in unselected patients with mCRPC was only moderate, there was a trend towards longer progression-free survival in patients with PTEN deletion^[75]^. In a study of hormone receptor-positive breast cancer patients treated with everolimus, patients with the PIK3CA/H1047R mutation had longer progression-free survival than patients with wild-type or other mutant forms of PIK3CA. This led the authors to suggest that the PIK3CA/H1047R mutation could be a potential biomarker of sensitivity to everolimus^[108]^. In the metastatic urothelial carcinoma setting, combination treatment with everolimus and the VEGF-TKI pazopanib showed significant clinical benefit in genomically selected patients with mutations in the mTOR pathway or FGFR^[109]^. An ongoing clinical trial is evaluating whether mutational status correlates with the response to a rapalog in colorectal cancer (NCT03439462)^[110]^. However, caution must be applied when selecting cancer patients for rapalog treatment based on their mutational profile. In a cohort of metastatic renal cell carcinoma patients, those who benefitted clinically from rapalogs more commonly had mutations in mTOR, TSC1 or TSC2 than patients who progressed. However, analysis of the extent and duration of response failed to suggest a correlation between truly exceptional responses and TSC1/TSC2/mTOR mutation. Indeed, 56% of responders had no mTOR pathway mutation identified^[111]^. Therefore, it appears that unlike, for example, lung cancer where there is a correlation between specific EGFR mutations and clinical responsiveness to EGFR tyrosine kinase inhibitors^[112]^, in many cases response to rapalogs is not so easy to define based on genotype.

### Promise of mTOR inhibitors as therapies to overcome resistance in patients refractory to other agents

Rapalogs have shown promise as second- and subsequent-line therapies in cancers that have become resistant to the targeting of other oncogenic pathways. Some early evidence for this came in the setting of RCC, where studies indicated that rapalog therapy following the development of VEGF receptor tyrosine kinase inhibitor (TKI) resistance (for example, progression on sunitinib) could be beneficial^[55,113]^. Indeed, the initial Food and Drug Administration approval for everolimus was for RCC where patients had progressed despite treatment with sunitinib, sorafenib, or both. The approval was based on the RECORD-1 trial (detailed in “The promise of mTOR inhibitors in cancer” section). Further evidence supports the use of everolimus in combination with another VEGF-targeted therapy, lenvatinib, with a high level of efficacy associated with the combined regimen in a Phase II study^[114]^ and anti-tumour activity also reported in a small Japanese cohort^[115]^. However, while everolimus is approved and effective in RCC, it may not be the only or optimal treatment for all patients. The INTORSECT trial found that second-line rapalog treatment in patients who had progressed following first-line VEGFR inhibition therapy did not demonstrate a progression-free survival advantage compared with treatment with an alternative VEGFR inhibitor^[116]^. This suggests that agents other than rapalogs may be equally or more effective in renal cell carcinoma that has progressed after first-line VEGFR TKI therapy^[117]^. Additionally, the risk-benefit balance needs to be considered, as RCC patients receiving lenvatinib plus everolimus reported more adverse events than those receiving either single-agent drug^[114]^.

Further evidence for the ability of rapalogs to show success in resistant cancers comes from studies of breast cancer patients. One study in endocrine-resistant hormone receptor(HR)-positive, HER2-negative metastatic breast cancer indicated that everolimus can potentially reverse resistance to endocrine therapies^[118]^. This supports earlier combination therapy studies in similar patient cohorts where the addition of everolimus to the treatment regime improved outcomes in patients who had shown aromatase inhibition resistance. In summary, these studies showed that: (1) combining everolimus with tamoxifen may reverse hormone resistance and lead to an increased clinical benefit rate, time to progression and overall survival compared with tamoxifen alone (TAMRAD trial)^[119]^; (2) the addition of everolimus to exemestane significantly improves progression-free survival (BOLERO-2)^[120]^; and (3) everolimus enhances the efficacy of fulvestrant (PrE0102 trial)^[121]^. Based on this evidence, everolimus was approved in 2012 to treat HR-positive, HER2-negative breast cancer patients. A further trial is now underway to determine whether concomitant administration of everolimus with endocrine therapy further prolongs progression-free survival in breast cancer patients who have not yet developed hormone resistance (Chloe trial)^[122]^. The recent MANTA Phase II trial is the first to report on the impact of adding the dual mTORC1/2 inhibitor, vistusertib (AZD2014), to endocrine therapy (fulvestrant) in an oestrogen-receptor positive advanced breast cancer cohort. Similar to the PrE0102 trial, MANTA demonstrated everolimus in combination with fulvestrant demonstrated significantly longer progression-free survival than fulvestrant alone^[123]^. However, the trial failed to show a benefit of adding the dual mTOR inhibitor to fulvestrant, despite pre-clinical promise. It remains to be investigated whether sub-optimal mTORC1 inhibition by vistusertib or some other factor could be responsible for the poor response seen^[123]^.

The results of trials using rapalogs as second-line therapy in other cancer types have been mixed. While little benefit of rapalog treatment has been seen in refractory gastric cancer^[124]^, rapalog treatment as a means of controlling refractory cancers has been seen more widely than just in RCC and breast cancer. For example, in an Italian Phase II study of patients with BTC which was progressing despite previous chemotherapy (EUDRACT 2008-007152-94), everolimus showed encouraging anti-tumour activity, with one patient demonstrating a complete response which was sustained for eight months^[125]^. Positive outcomes were also seen in thyroid cancer, where one trial aimed to determine whether the addition of everolimus to sorafenib is beneficial to patients who progress on sorafenib monotherapy (NCT01263951). Initial results indicate that the addition of everolimus allowed patients a median additional 13.7 months of disease stability^[126]^. Another active thyroid cancer trial (NCT00936858) indicates that everolimus therapy for patients who are radioactive iodine-refractory has significant benefits^[127]^. A Phase II study of everolimus with tivozanib in refractory colorectal cancer achieved stabilisation of disease in 50% of patients^[128]^. Evidence from the RADIANT-3 trial in PNET indicates that everolimus can prolong progression-free survival regardless of whether patients have had prior chemotherapy^[129]^.

## Conclusion

The targeting of mTOR signalling as a front-line therapy or as an alternative treatment to overcome resistance is of interest in many cancer types. While rapalogs may not have lived up to their initial promise suggested by *in vitro* data, they have had notable success in TSC and PNET, and are still actively being explored in other settings. With over 1000 clinical trials listed on clinicaltrials.gov involving the use of everolimus, temsirolimus or rapamycin in the cancer setting (as at November 2019), it is clear that substantial interest still exists in their clinical potential. As to the pitfalls of rapamycin resistance, steps are being taken to develop a new generation of mTOR inhibitors. The third generation, bivalent mTOR inhibitor, RapaLink, inhibits breast cancer cell growth at a level comparable to rapamycin, is effective against hyperactive mTOR-mutant cells and did not allow the evolution of resistance during the course of the study^[130]^. A follow-up study showed RapaLink-1 was also a potent inhibitor of both wild-type and active mTOR mutants in glioblastoma cell lines^[131]^. The pre-clinical *in vivo* aspect of the study showed RapaLink-1 had anti-tumour efficacy in both intracranial xenograft and spontaneously arising brain tumour models, indicating potential translational promise. However, a note of caution was that the initial tumour regression was followed by re-growth^[131]^. Therefore, the mechanisms behind this re-growth and whether this third generation of inhibitors will translate to the clinical setting remain to be evaluated. However, as the PI3K/Akt/mTOR signalling axis is one of the most commonly activated in human cancers, effective targeting of this pathway will remain of high interest to scientists and clinicians in their hunt for improved cancer therapies for patients.

## Declarations

### Copyright

© The Author(s) 2019.
